# Mammals in the MZNA Vertebrate Collection of University of Navarra, Spain

**DOI:** 10.3897/zookeys.634.10207

**Published:** 2016-11-21

**Authors:** Nora Escribano, David Galicia, Arturo H. Ariño, Carmen Escala

**Affiliations:** 1 Universidad de Navarra, Facultad de Ciencias, Department of Environmental Biology, Campus Universitario, 31080, Pamplona, Spain

**Keywords:** Mammals, occurrence, specimens, biometry, biogeographical regions, Iberian Peninsula

## Abstract

In this paper five datasets are described that provide information about records of mammals in the Vertebrate Collection of the Museum of Zoology of the University of Navarra (MZNA-VERT). The datasets contain 3,466 records belonging to 20 species of mammals sampled across the transition zone between the Atlantic and Mediterranean biogeographical regions (north Iberian Peninsula). The datasets include both distributional data (georeferenced records) and basic biometric data of most of the vouchered specimens stored in the museum facilities. The samples originated mainly within research projects and PhD theses carried out in the former department of Zoology and Ecology of the University of Navarra between 1982 and 2011. The Darwin Core Archive Format datasets are accessible through GBIF.

## Introduction

Natural History Museums collections are valuable worldwide as they have been documenting biodiversity for centuries, providing primary data for biodiversity research and conservation (Krishtalka and Humphrey 2000, Ponder et al. 2001). Data stored in museums have been used in a variety of studies investigating climate change, invasive species or changes in biodiversity (Shaffer et al. 1998, Suarez and Tsutsui 2004, Powney and Isaac 2015), but also in education, bringing science close to wider public (Jones 2013), although there are large amounts of locked data still waiting for digitization (Ariño 2010). Moreover, as museums often store sizable numbers of specimens, they are quite useful for taxonomical verification and reuse of metadata, which is most important for groups subject to systematic challenge (Berendsohn et al. 2010, Schilthuizen et al. 2015).

The Museum of Zoology of the University Navarra (MZNA) is a university museum founded in 1980 as the repository of fauna samples originated during research and instructional activities of the former Department of Zoology and Ecology (now integrated into the Department of Environmental Biology). The Museum is a data provider for the Global Biodiversity Information Facility (GBIF) and is an Affiliate to the International Commission on Zoological Nomenclature (ICZN). The Museum is also in charge of the curation and management of the Natural History Collections of the School of Sciences of the University of Navarra. MZNA stores more than two million specimens from a variety of taxa, from invertebrates to vertebrates (e.g. insects, springtails, nematodes, fish and mammals, among many other groups). In this paper we describe the datasets of mammals from the Vertebrate Collection of the MZNA that have been recently shared via GBIF, providing information about more than 3,000 accessions from direct captures and observations. Most of the collections consist of voucher-specimens, a specimen type that is crucial for verifying species identity (Rocha et al. 2014, Schilthuizen et al. 2015). The provenance area is an intensely sampled 10,000 km^2^ zone sitting astride three biogeographical regions, ideally suited for studies on biogeographical transitions and their shifts. Thus, the release of these data series would be of interest to anyone working on environmental change as they allow comparisons between current and past distributional patterns of the biodiversity of mammals at both local and global scales.

## General description

Purpose: the Vertebrate Collection of the Museum of Zoology of University of Navarra (2016) contains several subsets of data including mammals, birds and fish. We introduce here data compiled about mammals in the following datasets (Table 1):

Mammals in MZNA-VERT: project “Human impacts in rivers of Navarra” (AHER)

Mammals in MZNA-VERT: biology of *Arvicola sapidus* in Navarra. PhD project, Juan Manuel Garde (ARSA)

Mammals in MZNA-VERT: project “Loza” (BDLZ)

Mammals in MZNA-VERT: project “CAS” (CAS)

Mammals in MZNA-VERT: project “Biodiversity of mammals in Pamplona” (DVPA)

**Table 1.** Overview of the datasets.

Dataset	Records	Species	Latitude	Longitude	Temporal coverage	Sampling method	Measured specimens
AHER	2278	18	42.76 to 42.8	-1.52 to -1.39W	2001–2003	Trapping, pitfall, pellet analyses	212
ARSA	447	6	42.01 to 42.39N	-1.71 to -1.43W	1983–1992	Trapping, pellet analyses	321
BDLZ	220	8	42.83 to 42.84N	-1.73 to -1.71W	2007	Trapping	2
CAS	201	9	41.84 to 43.37N	-2.51 to -0.67W	1982–1983	Trapping	200
DVPA	342	15	42.76 to 42.87N	-1.73 to -1.56W	2011	Trapping, pellet analyses	2

## Project details

### Project citation

AHER: Actuaciones humanas en ríos de Navarra. Su incidencia en la conservación de la biodiversidad. Carmen Escala (Principal investigator, data collector), David Galicia (author).

ARSA: not receive funding.

BDLZ: not receive funding.

CAS: Efecto de la explotación y repoblación forestal sobre la fauna del suelo. Rafael Jordana (Principal investigator), Carmen Escala (author).

DVPA: Trabajo de campo para el estudio sobre las especies potenciales en la zona de Pamplona de especies de pequeños mamíferos. Carmen Escala (Principal investigator), David Galicia (author), Enrique Baquero (author).

Data digitization financial support: Acceso en línea a las colecciones de Ciencias Naturales de la Universidad de Navarra – I and II. Arturo H. Ariño (Principal investigator).

### Funding

The following institutions supported the projects that produced the field data compiled in MZNA-VERT: Gobierno de Navarra (AHER), Comisión Asesora de Investigación Científica y Tecnológica, Ministerio de Educación y Ciencia (CAS), Ayuntamiento de Pamplona (DVPA). In addition, two grants of the Ministry of Science and Education provided partial support for the data digitization (2005–2006 and 2009–2010).

### Study extent description

**Figure 1.**
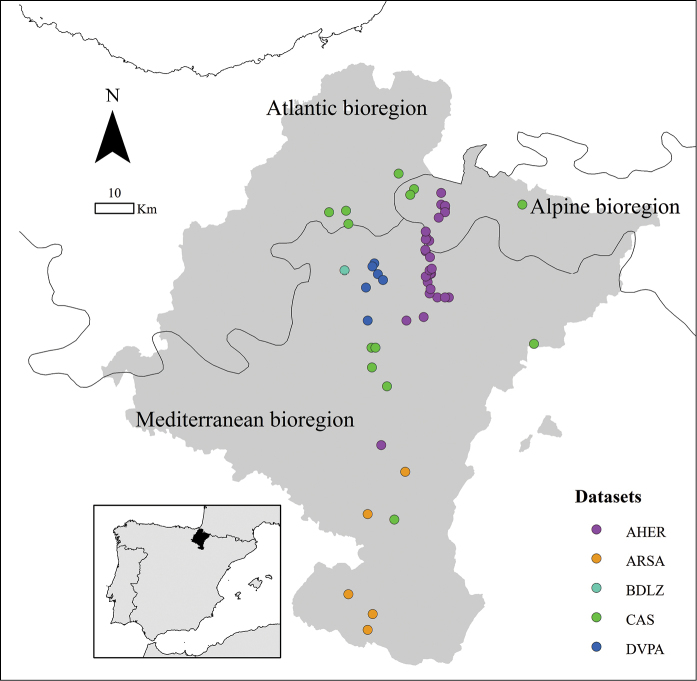
Study area: Navarra (inset, within Spain) and localities for the five datasets (AHER, ARSA, BDLZ, CAS, DVPA).

All data were collected in the province of Navarra, a 10,391 km^2^ region situated in the north of the Iberian Peninsula (Figure 1), between the western end of the Pyrenees and the Ebro basin. Location and topography ensure a wide range of local climates in Navarra, varying from the oceanic to mediterranean climate (Loidi and Báscones 1995). The northern half of Navarra belongs to the Eurosiberian region and the rest to the Mediterranean region, which is characterized by summer drought (Rivas-Martínez 1987). The high diversity of flora and fauna in Navarra derives from this sharp transition between bioregions over a relatively small distance of less than 160 km along a north-south axis (Loidi and Báscones 1995).

### Design description

The datasets compile all data provided by separate projects carried out in Navarra by research within the former department of Zoology and Ecology (now Environmental Biology). The resources of these datasets are research projects (BDLZ, CAS, DVPA) and PhD theses (AHER, ARSA).

## Taxonomic coverage

**Figure 2.**
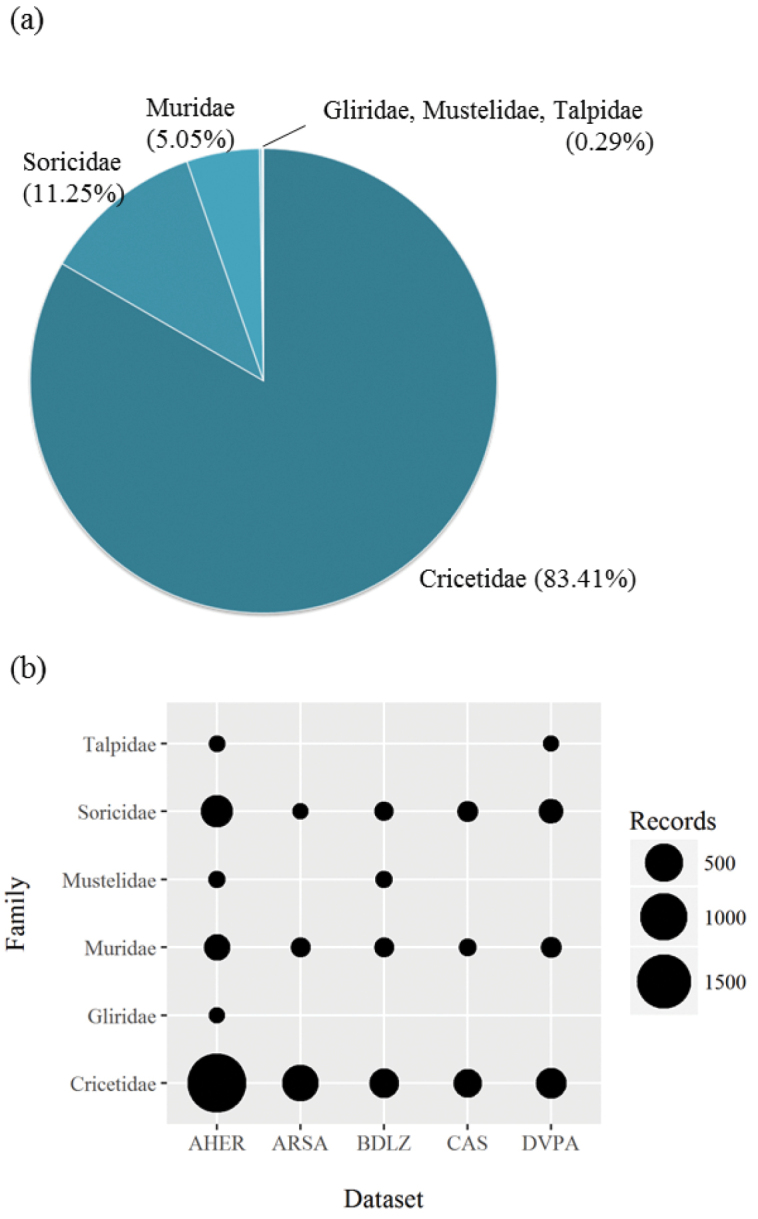
Taxonomic coverage of the datasets by families. Blob size proportional to number of records of each taxon in each dataset.

General taxonomic coverage: the datasets include information approximately 3,466 records of the orders Carnivora, Rodentia, and Soricomorpha (Figure 2). However, most of the datasets contain data of small mammals belonging to the families Soricidae and Cricetidae. Twenty species of small mammals are represented in the datasets.

### Taxonomic ranks

Kingdom: Animalia

Phylum: Chordata

Class: Mammalia

Order: Carnivora, Rodentia, Soricomorpha

Family: Cricetidae, Gliridae, Muridae, Mustelidae, Soricidae, Talpidae

Genus: *Apodemus*, *Arvicola*, *Crocidura*, *Eliomys*, *Microtus*, *Mus*, *Mustela*, *Myodes*, *Neomys*, *Rattus*, *Sorex*, *Suncus*, *Talpa*

Species: *Apodemus flavicollis* (Yellow-necked field mouse), *Apodemus sylvaticus* (Long-tailed field mouse), *Arvicola sapidus* (Southern water vole), *Crocidura russula* (Greater white-toothed shrew), *Eliomys quercinus* (Garden dormouse), *Microtus agrestis* (Field vole), *Microtus duodecimcostatus* (Mediterranean pine vole), *Microtus gerbei* (Pyrenean pine vole), *Microtus lusitanicus* (Lusitanian pine vole), *Mus domesticus* (House mouse), *Mus spretus* (Western Mediterranean mouse), *Mustela nivalis* (Least weasel), *Myodes glareolus* (Bank vole), *Neomys fodiens* (Eurasian water shrew), *Rattus norvegicus* (Brown rat), *Rattus rattus* (Black rat), *Sorex coronatus* (Millter’s shrew), *Sorex minutus* (Eurasian pygmy shrew), *Suncus etruscus* (Etruscan shrew), *Talpa europaea* (European mole).

## Spatial coverage

General spatial coverage: Navarra, north of Spain (Figure 1).

Coordinate box: 41°84'N to 43°37'N Latitude; -2°51'W to -0°67'W Longitude.

## Temporal coverage

**Figure 3.**
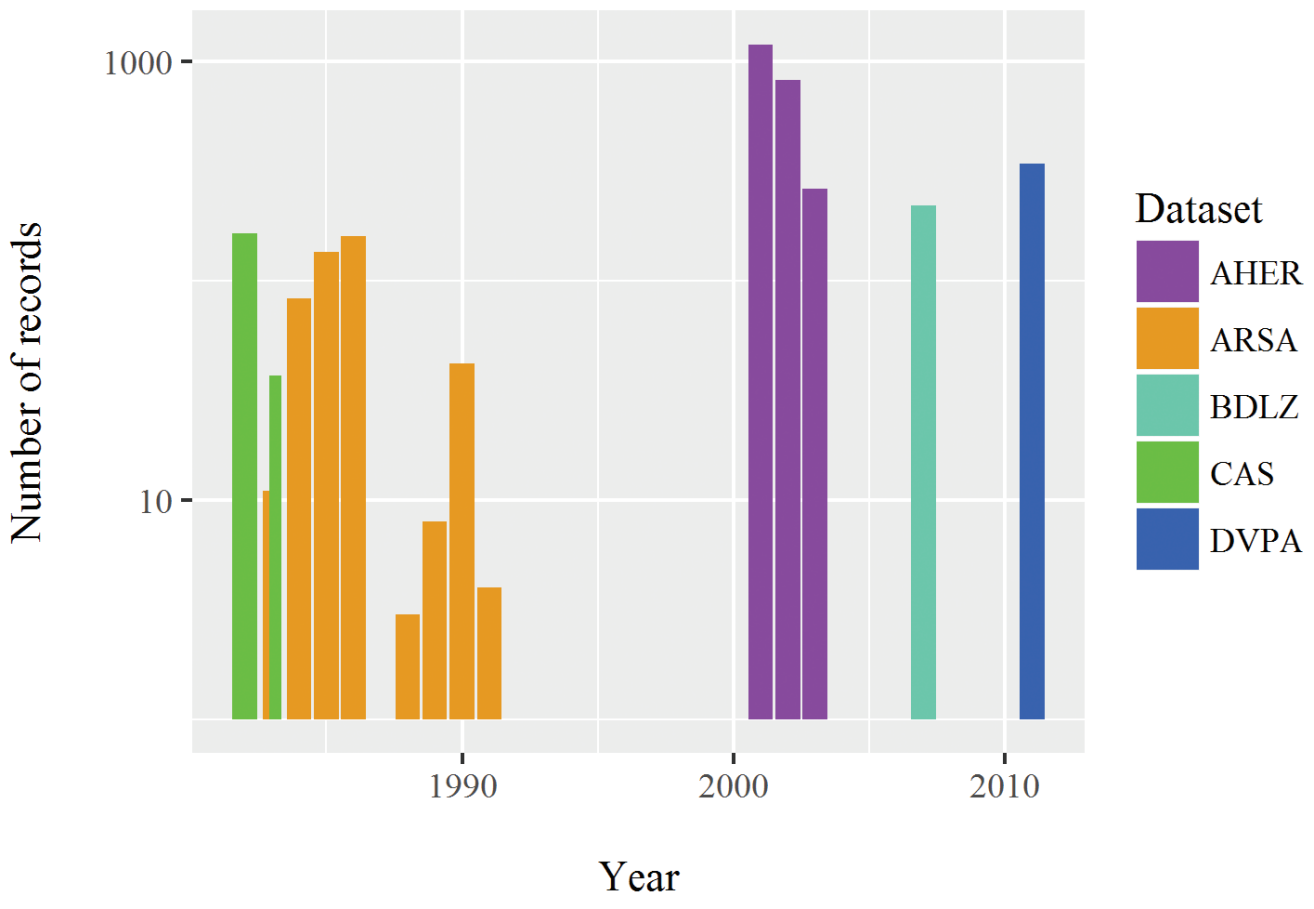
Temporal distribution of the records by dataset. Number of records in log scale.

1982-2011 (see Figure 3).

## Methods

All projects were carried out in different areas of Navarra (Spain, Figure 1) and under different sampling designs (see sampling description per dataset).

### Study extent per dataset

AHER: The Erro river basin is located in the north of Navarra in the western side of the Pyrenees. Its climate conditions strongly vary from the source of the Erro River, influenced by Pyrenean climate, to its mouth river, where the Mediterranean climate rules. Rainfall decreases while temperature increases from north to south. The river traverses a wide range of landscapes, which have a positive effect on the flora and fauna diversity. The headwater’s area and the middle section of the basin are dominated by oak and beech forests, pastures and forestry exploitation mostly consisting of pine stands. The lower section is dominated by crops and cattle grazing fields (Rivas-Martínez 1987).

ARSA: Ribera Alta is a geographic area located in the south of Navarra dominated by an agricultural landscape mixed with natural vegetation coverage consisting mainly of Mediterranean scrub patches and black pine repopulations (Rivas-Martínez 1987). Surveys took place at the Aragón's riverbank as it passes through the Mélida municipality. The bank has a riparian forest including elms, black poplars, ashes and willow trees. Reed beds in the area provide shelter and a source of food for the water vole.

BDLZ: The Loza pond is situated in Pamplona basin in the centre of Navarra. Pools and ponds play an important role in biodiversity conservation as they afford a stopover plot for migrant birds. Loza stands out as being one of the few semi-natural still water environments of the northern sector in Navarra, and makes one of the first rest places for incoming birds from the north of Europe through the west Pyrenees route. Land cover in Loza is characterised by prairies, scrubland, reed bed patches and some black poplar as well. Crops and pine repopulations enclose the pond (Arizaga et al. 2009).

CAS: This collection is part of a wider project that encompasses the study of soil fauna. Here, only the information related to mammal samplings is reported. In each locality one or two types of habitats were studied: Bardenas Reales (Mediterranean maquis and Aleppo pine stands), Sansoain (holm oak forest and black pine repopulation), Carrascal (Mediterranean scrub), Bigüezal (Scots pine forest and meadow), Beunza (Japanese larch stands), Quinto Real (Japanese larch stands and meadow) and Irati (beech forest).

DVPA: Pamplona, pop. approx. 200,000, is a city located in the middle of Navarra. The urban area includes several parks which constitute a refuge for wild fauna, especially birds and small mammals. Pamplona is traversed by the Arga River which plays a key role as a natural corridor for fauna. Six sampling locations were chosen to study the small mammal community of the wider Pamplona municipality. Azoz and Zolina are two small villages near Pamplona that were checked for pellets. Azoz is situated in the north near Ezcaba Mountain while Zolina lies to the south of the city. Like Pamplona they are surrounded by crop fields although there are hills covered by pines and oaks. Trapping campaigns took place in the four main biotopes that can be found in Pamplona and its surroundings: riparian forest of Arga River, pine repopulation, Mediterranean scrubland and crop field.

### Sampling description per dataset

AHER: The project was conducted between 2001 and 2003. Fifteen sites were sampled seasonally during two consecutive years. Sherman traps (7.5×9×23 cm) baited with bread and oil were used. Each sampling event consisted of two nights with an average sampling effort of 160 traps per night. Specimens were sexed and identified in the field when possible. Some of the specimens captured were transported to the laboratory for further studies. In order to reduce the bias associated with single-type sampling method, eleven sites were additionally sampled using pitfall traps in order to get a better knowledge of the small mammal community. Each sampling site was composed of six cylindrical jars (11.5 cm diameter and 13.5 cm depth) which were active from May to November 2001. Finally, Barn owl pellets, information about footprints and other mammal trails were collected during the sampling period.

ARSA: The main sampling effort occurred from October 1984 to December 1986 once or twice a month. Five sampling sites were selected but most of the material comes from Mélida, specifically from the riparian forest around the Aragón river. Traps were placed in areas where activity of *Arvicola sapidus* was observed (e.g. scats). Each trapping event lasted one day and traps were checked twice a day, at dawn and twilight. Traps were not baited to avoid attracting other species. All specimens captured were brought to the laboratory for further studies.

BDLZ: Sampling was conducted between February and October in 2007 and consisted of live-trapping sessions. Sherman traps (7.5×9×23 cm) were placed along a habitat gradient varying from pastures to scrubland following a line-transect. The distance between traps was approximately 10 metres. Only half of the traps were baited in order to avoid bias due to the baiting. Each sampling event lasted four nights. Traps were placed on the first night and checked for animals during the next four days. Total sampling effort was 800 traps-night. Specimens were sexed, identified in the field using external morphology and then released. In addition, a few barn owl pellets were collected within the study area during the sampling period.

CAS: Sampling took place between 1982 and 1983. Snap traps were used for sampling the small mammal community. They were active for 15 days. All captured specimens were brought to the laboratory for further studies.

DVPA: The project was carried out in 2011 and consisted of live-trapping samplings and barn owl pellet analyses. Live trapping campaigns were conducted in four locations from June to September (2011). Sherman traps (7.5×9×23 cm) were baited with bread and oil and placed along a line-transect. Each sampling event consisted of two nights and the average sampling effort made was 80 traps per night. Specimens were sexed and identified in the field and then released. Specimens found dead were brought to the laboratory. Barn owl pellets were found in Azoz and Zolina. Churches and barns were checked for barn owl pellets in these villages. When found, they were transported to the laboratory for later identification of the remains of prey items.

### Method description

All data provided by the different projects were systematically incorporated to MZNA database (Ariño 1991) and given unique catalogue numbers.

In the case of data obtained from the analyses of barn owl pellets (in AHER, BDLZ, and DVPA datasets), all pellets were processed as follows: pellets were frozen at -20°C for bug removal. Afterwards, they were dissected separating skulls, mandibles and other bones from the rest of remains (e.g. fur, broken bones or insect’s remains). Specimens were identified using a stereoscopic microscope and appropriate literature (Gosálbez 1987). Finally, specimens were placed in zip plastic bags with their unique catalogue number and stored in the museum facilities.

All datasets involved trapping surveys. Most specimens were brought to the laboratory for further studies (AHER, ARSA, CAS) or released except for individuals found already dead (BDLZ, DVPA). Some specimens from the datasets were measured and prepared in the laboratory (Table 1). Measurements were taken following rules from Comisión de Biometría (1972). Next, the skin of dead individuals was removed, cleaned with soap and dried using sodium borate, mounted on cardboard and stored in MZNA facilities labeled with their unique catalogue number. Afterwards, skull, mandibles and in some cases skeleton parts were obtained and preserved dry in zip-lock plastic bags with their unique catalogue number. Generally, after preparing the skin, specimens were boiled and flesh was separated from the bones, also using a KOH 0.1% solution if needed. ARSA dataset also contains tissues (e.g. crystalline, testicles, ovaries and histological preparations in slides) conserved in ethanol (ETOH 70%) from specimens of *Arvicola sapidus*.

### Quality control description

All specimens were deposited in the Museum of Zoology of the University of Navarra (MZNA, Pamplona, Spain) within its Vertebrate Collection.

All the species were sexed and identified in the field (when possible) and the taxonomic identity of each specimen brought to the laboratory was verified by experienced researchers using suitable literature (Gosálbez 1987).

All datasets have been standardized to Darwin Core standards. First, we checked errors and inconsistencies in the data following the guidelines by Chapman (2005). Scientific names were checked and synonyms exchanged for valid names according to Mammal Species of the World and the atlas of mammals from the Iberian Peninsula (Wilson and Reeder 2005, Palomo et al. 2007). All coordinates in UTM/MGRS were transformed to the geographic system. The uncertainty of coordinates was calculated in metres using the point-radius method. Finally, we checked locality consistency by visual inspection overlapping them against an administrative map of Navarra using GIS(ESRI 2015). All doubtful records were checked and corrected.

## Data resources

The datasets are deposited at GBIF, the Global Biodiversity Information Facility, MZNA (2016) Mammals in MZNA-VERT: project “Human impacts in Navarra’s rivers”. v1. University of Navarra, Museum of Zoology. Dataset/Occurrence. http://www.gbif.es/ipt/resource?r=mzna_vert_mast_aher&amp;v=2.1 http://doi.org/10.15470/gzw8bz

MZNA (2016) Mammals in MZNA-VERT: biology of *Arvicola sapidus* in Navarra. PhD project, Juan Manuel Garde. v1. University of Navarra, Museum of Zoology. Dataset/Occurrence. http://www.gbif.es/ipt/resource?r=mzna_vert_mast_arsa&amp;v=1.0 http://doi.org/10.15470/zqgojj

MZNA (2016) Mammals in MZNA-VERT: project “Loza”. v1.2. University of Navarra, Museum of Zoology. Dataset/Occurrence. http://www.gbif.es/ipt/resource?r=mzna_vert_mast_bdlz&amp;v=1.2 http://doi.org/10.15470/iquxkg

MZNA (2016) Mammals in MZNA-VERT: project “CAS”. v1.1. University of Navarra, Museum of Zoology. Dataset/Occurrence. http://www.gbif.es/ipt/resource?r=mzna_vert_mast_cas&amp;v=1.1 http://doi.org/10.15470/swjzkt

MZNA (2016) Mammals in MZNA-VERT: project “Biodiversity of mammals in Pamplona”. v1. University of Navarra, Museum of Zoology. Dataset/Occurrence. http://www.gbif.es/ipt/resource?r=mzna_vert_mast_dvpa&amp;v=1.0 http://doi.org/10.15470/6dxd1d

## Datasets descriptions

Object name

Darwin Core Archive Mammals in MZNA-VERT: project human impacts in rivers of Navarra

Darwin Core Archive Mammals in MZNA-VERT: biology of *Arvicola sapidus* in Navarra. PhD project. Juan Manuela Garde

Darwin Core Archive Mammals in MZNA-VERT: project Loza

Darwin Core Archive Mammals in MZNA-VERT: project CAS

Darwin Core Archive Mammals in MZNA-VERT: project biodiversity of mammals in Pamplona

Character encoding: UTF-8

Format name: Darwin Core Archive format

Format version: AHER (v2.1), ARSA (v1.0), BDLZ (1.2), CAS (v1.1) and DVPA (v1.0).

Distribution: all datasets can be found in http://datos.gbif.es/collectory/public/show/co82

Publication date of data: 2016-03-22 (ARSA, DVPA), 2016-04-14 (AHER, BDLZ, CAS).

Language: English

Licenses of use: These datasets are made available under a Creative Commons Attribution Non Commercial (CC-BY-NC) 4.0 License

Date of metadata creation: 2016-03-22 (AHER, ARSA, CAS, DVPA), 2016-04-06 (BDLZ)

Hierarchy level: Dataset
